# Pathogenesis of Neovascular Glaucoma in Diabetic Retinopathy: A Review

**DOI:** 10.1155/joph/6448312

**Published:** 2026-06-29

**Authors:** Xuan Wang, Siyan Liu, Qi Wu, Yuxin Lv, Shuangshuang Yi, Bowen Shi, Zhengzheng Wu, Jing Yan

**Affiliations:** ^1^ China Academy of Chinese Medical Sciences Eye Hospital, No. 33 Lugu Street Shijingshan District, Beijing, 100040, China

**Keywords:** diabetic retinopathy, epigenetics, neovascular glaucoma, oxidative stress, pathogenesis, targeted therapy

## Abstract

Neovascular glaucoma (NVG) secondary to diabetic retinopathy (DR) is a severe complication driven by ischemia‐induced angiogenesis. Current evidence indicates that the pathogenesis begins with retinal hypoxia caused by hyperglycemia‐induced capillary occlusion, which stabilizes hypoxia‐inducible factor‐1α (HIF‐1α) and upregulates vascular endothelial growth factor (VEGF). VEGF‐A and placental growth factor (PlGF) drive abnormal angiogenesis in the retina, iris, and anterior chamber angle. Downregulation of endogenous inhibitors such as pigment epithelium‐derived factor (PEDF) may exacerbate this process. Concomitant inflammation mediated by cytokines including interleukin‐6 (IL‐6) and tumor necrosis factor‐α (TNF‐α), followed by transforming growth factor‐β (TGF‐β)‐induced fibrotic angle closure, collectively leads to refractory intraocular pressure elevation and optic nerve damage. Clinical outcomes are influenced by genetic polymorphisms, renal comorbidities, and the aqueous humor biomarker profile. Current anti‐VEGF monotherapy is limited by its inability to control fibrosis and inflammation, highlighting the need for multifaceted therapeutic strategies incorporating anti‐inflammatory and antifibrotic agents. Critical knowledge gaps remain in longitudinal human data and preclinical models. To improve the prognosis of this devastating disease, shifting toward systemic metabolic management and prospective multiomics‐based risk stratification may represent future directions.

## 1. Introduction

Neovascular glaucoma (NVG) is a severe complication of proliferative diabetic retinopathy (PDR), characterized by refractory intraocular pressure elevation, anterior segment fibrovascular proliferation, and rapidly progressive optic nerve degeneration. Despite advances in antiangiogenic pharmacotherapy, NVG remains a leading cause of irreversible blindness due to delayed diagnosis and an incomplete understanding of its multifactorial pathogenesis. This review synthesizes current evidence to systematically delineate the mechanistic cascade from chronic hyperglycemia to NVG, with a focus on clinically relevant pathways beyond angiogenesis.

Retinal ischemia—resulting from microvascular occlusion and capillary nonperfusion—is considered the primary trigger, stabilizing hypoxia‐inducible factor‐1α (HIF‐1α) and upregulating vascular endothelial growth factor (VEGF) and placental growth factor (PlGF). These mediators drive neovascularization spreading from the posterior pole to the iris and anterior chamber angle [1, 2]. However, VEGF alone cannot account for the fibrotic contraction and structural collapse that limit the efficacy of anti‐VEGF monotherapy [3, 4]. Concomitant inflammation, mediated by macrophage infiltration, IL‐6, and TNF‐α, increases vascular permeability and facilitates cytokine transport into the aqueous humor [5]. Critically, transforming growth factor‐β (TGF‐β) is recognized to drive myofibroblast differentiation and extracellular matrix remodeling, ultimately leading to PAS and irreversible angle closure [3]. These interrelated processes—hypoxia, angiogenesis, inflammation, and fibrosis—collectively disrupt aqueous humor outflow and induce secondary optic neuropathy through both mechanical and neurodegenerative mechanisms.

The clinical heterogeneity of NVG progression reflects the influence of genetic variations, systemic comorbidities, and emerging biomarkers. NVG should be regarded not only as an ophthalmic emergency but also as an end‐stage manifestation of systemic metabolic dysregulation. This review integrates multimodal therapeutic paradigms that combine antiangiogenic, anti‐inflammatory, and antifibrotic strategies. Ultimately, shifting clinical focus toward “pre‐NVG” detection—defined as a stage before the appearance of iris or angle neovascularization when only aqueous humor biomarker abnormalities or subtle imaging changes are present—using advanced imaging and molecular profiling may enable proactive intervention before irreversible structural damage occurs.

## 2. Methods

We systematically searched the peer‐reviewed literature in PubMed, MEDLINE, and Embase databases on DR, hyperglycemia, and microvascular complications. The search strategy combined MeSH/Emtree terms with keywords such as endothelial dysfunction, pericyte loss, metabolic memory, and oxidative stress. Inclusion criteria were limited to randomized controlled trials, landmark clinical studies (e.g., Diabetes Control and Complications Trial/Epidemiology of Diabetes Interventions and Complications, DCCT/EDIC), and high‐quality mechanistic studies published in English within the past two decades. Reviews, editorials, and conference abstracts were excluded. Relevant references were manually screened to ensure comprehensiveness of the evidence base.

## 3. Mechanisms

### 3.1. Overall Pathophysiological Cascade: From Hyperglycemia to Optic Atrophy

**FIGURE 1:**
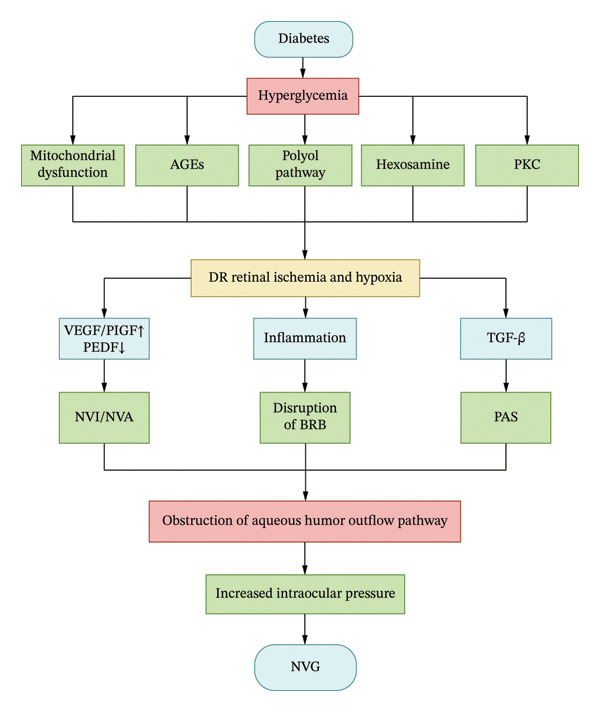
Pathogenic mechanism of diabetes‐induced NVG. Diabetes leads to persistent hyperglycemia, which directly triggers mitochondrial dysfunction and activates four key biochemical pathways: accumulation of aAGEs, polyol pathway activation, hexosamine pathway activation, and overexpression of PKC. These pathological changes jointly induce retinal ischemia and hypoxia in DR. Hypoxia further upregulates the expression of VEGF/PlGF and downregulates PEDF, promoting the formation of NVI/NVA. Meanwhile, metabolic disorders caused by hyperglycemia initiate inflammatory responses, disrupting the BRB. Additionally, TGF‐β is activated, leading to the formation of PAS. Ultimately, NVI/NVA, BRB disruption, and PAS work together to obstruct the aqueous humor outflow pathway, resulting in increased intraocular pressure and the progression to NVG. NVG: neovascular glaucoma; AGEs: advanced glycation end products; PKC: protein kinase C; DR: diabetic retinopathy; VEGF: vascular endothelial growth factor; PlGF: placental growth factor; PEDF: pigment epithelium‐derived factor; NVI: neovascularization of the iris; NVA: neovascularization of the angle; BRB: blood‐retinal barrier; TGF‐β: transforming growth factor‐β; PAS: peripheral anterior synechiae.

Chronic hyperglycemia in diabetes activates five classic metabolic pathways—mitochondrial dysfunction, advanced glycation end‐product (AGE) formation, polyol pathway flux, hexosamine pathway activation, and protein kinase C (PKC) activation. These pathways collectively induce retinal ischemia and hypoxia, which serve as the central trigger for NVG. Retinal ischemia/hypoxia subsequently drives three interrelated pathological axes: (1) Angiogenic imbalance, characterized by upregulation of VEGF and PlGF alongside downregulation of pigment epithelium‐derived factor (PEDF), leading to neovascularization of the iris (NVI) and the anterior chamber angle (NVA). (2) Inflammation, which disrupts the blood‐retinal barrier (BRB) and increases vascular permeability. (3) TGF‐β‐mediated fibrosis, which results in peripheral anterior synechiae (PAS) and angle closure. These three processes synergistically obstruct the aqueous humor outflow pathway, causing sustained intraocular pressure (IOP) elevation and ultimately optic atrophy. The following subsections detail the molecular mechanisms of each step, while Figure 1 provides a schematic overview of this core pathogenic cascade.

### 3.2. Retinal Ischemia Drives NVG

#### 3.2.1. Microvascular Dysfunction and Capillary Occlusion

Chronic hyperglycemia leads to pericyte and endothelial cell loss, causing capillary wall fragility, microaneurysm formation, and nonperfusion. These changes arise from activation of the polyol pathway, PKC, and hexosamine pathway and accumulation of AGEs6. AGE‐RAGE binding triggers NF‐κB‐mediated inflammation, upregulates ICAM‐1, promotes leukostasis, and results in capillary occlusion [6, 7]. High glucose induces mitochondrial fragmentation and dysfunction, further compromising endothelial integrity [8]. Clinically, impaired vascular reactivity and reduced ocular blood flow precede overt signs of DR [9]. Microvascular dysfunction may actively drive ischemia toward NVG progression.

#### 3.2.2. Hypoxia‐Induced HIF‐1α Activation

Capillary occlusion causes retinal hypoxia, which stabilizes HIF‐1α. HIF‐1α then translocates to the nucleus and activates proangiogenic genes such as VEGF1. HIF‐1α levels strongly correlate with VEGF levels in PDR patients, and HIF‐1α knockdown inhibits neovascularization [10]. HIF‐1α also upregulates erythropoietin, PlGF, and IL‐6, sustaining abnormal vascular growth [11]. Even under mild hypoxic conditions, ROS can stabilize HIF‐1α, amplifying hypoxic signaling [12]. HIF‐1α converts ischemia into neovascular signals, leading to anterior segment neovascularization and secondary angle closure.

#### 3.2.3. Hypoxia Predicts Neovascular Risk

Clinical studies using direct oxygen measurement have shown [13] that prereinal pO_2_ in PDR eyes is below 10 mmHg and correlates with elevated vitreous levels of VEGF, IL‐6, and TNF‐α. A retrospective cross‐sectional study using ultra‐widefield angiography found that patients with peripheral nonperfusion areas larger than 5 disc diameters had a 5‐fold increased risk of anterior segment neovascularization [14]. Another clinical study suggested that a long axial length is associated with lower oxygen consumption and reduced PDR risk [15]. Animal experiments have demonstrated that eyes with > 30% reduction in blood flow in mouse models exhibit increased capillary occlusion [9]. Together, these findings indicate that local tissue hypoxia is closely linked to neovascular risk and may provide an important basis for risk stratification in PDR.

### 3.3. VEGF‐Mediated Angiogenesis and Anterior Segment Diffusion

#### 3.3.1. VEGF‐A and PlGF Synergistically Drive Retinal and Iris Neovascularization

VEGF‐A and PlGF are considered major drivers of pathological neovascularization in PDR [16]. Elevated vitreous VEGF‐A levels are strongly associated with the severity of retinal neovascularization, and clinical studies have confirmed that VEGF‐A expression in the vitreous of PDR patients is significantly higher than that in nondiabetic controls [17]. As a specific ligand for VEGFR‐1, PlGF can synergize with VEGF‐A to amplify proangiogenic signaling pathways [18]. Unlike VEGF‐A, PlGF is nearly undetectable in the normoxic retina but is significantly upregulated under ischemic conditions; clinical studies have shown that PlGF levels in the aqueous humor of PDR patients are markedly elevated and decline significantly after anti‐VEGF therapy [19]. Moreover, PlGF not only enhances VEGF‐A‐induced vascular permeability but also recruits bone marrow‐derived macrophages and promotes the secretion of proinflammatory cytokines, establishing a positive feedback loop between inflammation and neovascularization that further drives disease progression [18].

#### 3.3.2. Failure of Endogenous Antiangiogenic Defense Mechanisms

NVG progression results not only from excessive proangiogenic signaling but also from the failure of endogenous inhibitors. PEDF, secreted by the retinal pigment epithelium, is a key endogenous antiangiogenic factor that maintains retinal vascular homeostasis and possesses multiple biological functions including antiangiogenesis, antioxidation, anti‐inflammation, and neuroprotection [20]. Under physiological conditions, PEDF maintains retinal vascular quiescence by antagonizing VEGF activity [21]. In PDR, hyperglycemia and oxidative stress downregulate PEDF expression through multiple pathways, disrupting the PEDF‐VEGF balance and thereby releasing the molecular brake on neovascularization [22].

PEDF exerts its antiangiogenic effects by inducing apoptosis of activated endothelial cells, inhibiting endothelial cell migration, and maintaining BRB integrity [20]. Clinical studies have found that vitreous PEDF levels are significantly lower in PDR patients than in nondiabetic controls, while VEGF levels are markedly higher; this imbalance is closely associated with neovascular activity [23]. Furthermore, accumulation of AGEs under hyperglycemic conditions can further inhibit PEDF expression and upregulate VEGF levels through induction of oxidative stress, creating a vicious cycle [22]. Therefore, the PEDF‐VEGF imbalance may represent a critical link in the transition from vascular homeostasis to uncontrolled angiogenesis.

#### 3.3.3. Mechanisms of Cytokine Diffusion From the Posterior to the Anterior Segment

The diffusion of neovascular stimuli from the ischemic retina to the anterior segment depends on the biophysical properties of the vitreous and the molecular characteristics of the cytokines. The vitreous is a gel‐like matrix composed of collagen fibers and hyaluronic acid; its structural integrity is altered in the diabetic state, which can affect cytokine diffusion efficiency [24]. Both VEGF‐A and PlGF are small molecular weight proteins that can diffuse through the extracellular matrix [25]. Cytokines that enter the anterior chamber bind to VEGFR‐2 on the vascular endothelium of the iris and ciliary body, triggering iris neovascularization [26].

Clinical imaging studies have shown that the extent of peripheral retinal ischemia is closely associated with the risk of anterior segment neovascularization, suggesting that cumulative cytokine exposure exceeding a pathogenic threshold is a key step in the development of iris neovascularization [27].

It should be noted that, in addition to vitreous diffusion, the vascular systems of the retina, iris, and ciliary body also participate in this process: the ischemic retina is the source of cytokines, while the iris and ciliary body vessels are the target tissues, and the degree of VEGFR‐2 expression on their endothelium may determine the intensity of the neovascular response [26]. As the only fluid medium connecting the posterior and anterior segments, the vitreous diffusion efficiency is a critical rate‐limiting step determining the exposure level of the anterior segment. The rapid regression of iris neovascularization after intravitreal anti‐VEGF injection confirms the dynamic equilibrium of cytokine distribution [26]. The vitreous is not a static barrier but a dynamic channel for the spatial diffusion of angiogenic signals. Cytokines diffusing into the anterior chamber first induce iris neovascularization; subsequently, fibrovascular membranes undergo fibrotic contraction, pulling the iris toward the corneal endothelium or trabecular meshwork, forming PAS, and ultimately mechanically closing the angle, leading to NVG. Therefore, the complete pathological steps from cytokine diffusion to angle closure can be summarized as follows: cytokine diffusion leads to iris neovascularization, followed by fibrovascular membrane formation and contraction, development of PAS, and finally angle closure.

### 3.4. Inflammatory Amplification Beyond Angiogenesis

#### 3.4.1. Innate Immune Activation

In the diabetic retina, circulating monocytes are recruited to ischemic areas and differentiate into M1 macrophages, releasing various inflammatory mediators that may exacerbate BRB breakdown. In diabetic rodent models, retinal macrophages aggregate around areas capillary nonperfusion [28].

Resident microglia undergo morphological transformation in response to metabolic stress, become activated via the Toll‐like receptor 4/NF‐κB signaling axis, and release cytokines such as TNF‐α and IL‐1β. These act synergistically with proangiogenic factors to aggravate BRB disruption and pathological neovascularization [29]. In the human PDR retina, activated microglia colocalize with Müller cells, potentially forming an inflammatory microenvironment that sustains neuroinflammation [30]. Under hypoxic conditions, microglia drive retinal angiogenesis through the HMOX1‐STAT3 signaling axis [31].

#### 3.4.2. Aqueous Humor Cytokines as Clinical Biomarkers

As DR progresses, inflammatory mediators diffuse into the anterior chamber, forming a detectable cytokine gradient in the aqueous humor. A meta‐analysis has shown that levels of inflammatory factors such as IL‐6 and TNF‐α in the aqueous humor are significantly elevated in diabetic patients [30]. Clinical studies have confirmed that concentrations of VEGF‐A, PlGF, IL‐1β, IL‐23, IL‐17A, and TNF‐α in the aqueous humor of PDR patients are significantly higher than those in nondiabetic controls [19]. In eyes with anterior segment neovascularization, these factors are further elevated and strongly correlate with the severity of anterior segment pathology [19]. TNF‐α upregulates E‐selectin and ICAM‐1, promoting leukocyte adhesion to iris vessels, while IL‐6 participates in Th17 cell differentiation, exacerbating local inflammation [32]. Moreover, elevated levels of sICAM‐1 in the aqueous humor indicate a sustained state of vascular endothelial activation, reflecting the degree of BRB breakdown [32]. Dynamic monitoring of aqueous humor cytokines can provide important biomarkers for early diagnosis, disease staging, and treatment evaluation in DR [31].

### 3.5. Fibrotic Transformation and Structural Collapse of the Angle

#### 3.5.1. TGF‐β‐Mediated Myofibroblast Differentiation in the Iris and Trabecular Meshwork

TGF‐β plays a key role in the fibrotic progression of NVG by inducing differentiation of iris and trabecular meshwork cells into myofibroblasts [33]. Concentrations of TGF‐β1 and TGF‐β2 in the aqueous humor of diabetic patients are significantly elevated and positively correlate with HbA1c levels [34]. Elevated TGF‐β activates the Smad2/3 signaling pathway, leading to upregulation of α‐smooth muscle actin (α‐SMA) expression and stress fiber formation, thereby driving myofibroblast differentiation [35]. In animal models, TGF‐β2‐induced fibrotic reactions result in a marked increase of α‐SMA‐positive cells in the trabecular meshwork and reduced aqueous humor outflow facility [36]. In human NVG eyes, the trabecular meshwork and posterior iris surface exhibit intense α‐SMA immunoreactivity, and contraction of the fibrovascular membrane pulls the iris toward the cornea, initiating PAS formation [37]. Mechanical stress amplifies TGF‐β‐mediated fibrosis through the integrin‐focal adhesion kinase signaling pathway, potentially creating a self‐sustaining positive feedback loop that further drives disease progression [38].

#### 3.5.2. Extracellular Matrix (ECM) Remodeling

Myofibroblasts drive pathological ECM remodeling through excessive collagen synthesis and an imbalance between synthesis and degradation. In PDR, the TGF‐β/SMAD signaling pathway is activated, promoting fibrovascular membrane formation on the retinal and iris surfaces and leading to upregulation of collagens (COL1a1, COL4a1) and α‐SMA (ACTA1) in the trabecular meshwork [39]. Studies in diabetic mouse eyes have shown that upregulated TGF‐β2 expression in the anterior segment leads to increased ECM deposition in the trabecular meshwork and thickening of the scleral and retinal capillary basement membranes, suggesting that early DR‐related structural changes occur independently of the VEGF pathway [40]. The balance between matrix metalloproteinases (MMPs) and their tissue inhibitors (TIMPs) is crucial for maintaining ECM homeostasis. In the vitreous of PDR patients, TIMP2 levels and the TIMP2/MMP2 ratio are significantly elevated, and the presence of diabetes and preoperative glaucoma is significantly associated with higher TIMP1 levels, indicating that MMP/TIMP imbalance plays an important role in DR‐related dysfunction of the aqueous humor outflow pathway [41].

Lysyl oxidase (LOX) and LOXL2‐mediated ECM cross‐linking enhance tissue stability and affect aqueous humor outflow resistance. TGF‐β2 induces upregulation of LOX and LOXL2 expression in trabecular meshwork cells; these enzymes covalently cross‐link ECM proteins, rendering them more resistant to degradation and thereby increasing outflow resistance [42]. In diabetic eyes, AGEs form nonenzymatic cross‐links with collagen, disrupting vascular basement membrane structure and further exacerbating tissue stiffening and fibrosis progression [43]. In summary, activation of the TGF‐β signaling pathway, MMP/TIMP imbalance, LOX‐mediated cross‐linking, and AGE accumulation collectively drive pathological ECM remodeling in the diabetic state, ultimately impairing the function of the aqueous humor outflow pathway.

#### 3.5.3. Irreversible PAS Formation

The terminal stage of fibrotic transformation is the formation of irreversible PAS, in which the iris becomes permanently adherent to the cornea or trabecular meshwork, resulting in severe compromise of the aqueous humor outflow pathway. Clinically, PAS is a key marker of progression to the irreversible stage of NVG, and its presence is closely associated with the development of NVG and poor visual prognosis [44].

Histopathologically, myofibroblasts within the fibrovascular membrane drive traction of the iris toward the cornea through their contractile properties, initiating and exacerbating PAS formation [45]. Once PAS form, impaired aqueous humor outflow leads to sustained IOP elevation, which further aggravates retinal ischemia, creating an ischemia‐fibrosis vicious cycle [46]. Once extensive PAS develop, spontaneous resolution is extremely rare, and clinical management relies on surgical interventions such as goniosynechialysis or drainage valve implantation to re‐establish aqueous humor outflow [47]. Therefore, preventing or delaying the onset and progression of PAS is a key strategy for the management of NVG.

### 3.6. IOP Dysregulation and Secondary Optic Neuropathy

#### 3.6.1. Obstructive Mechanisms

The elevated IOP in secondary glaucoma arises from three interrelated mechanical outflow obstruction mechanisms. PAS are fibrovascular adhesions between the peripheral iris and the angle, which can physically occlude the trabecular meshwork structure, leading to impaired aqueous humor outflow [48]. Patients with a larger extent of PAS on gonioscopy often exhibit medically refractory IOP elevation and poor surgical outcomes [49]. Iris bombe results from an annular pupillary block that traps aqueous humor in the posterior chamber, forcing the peripheral iris to bow forward against the angle structures, further exacerbating angle closure [48]. Trabecular meshwork obstruction involves the invasion of fibrovascular tissue into the trabecular meshwork spaces, where deposited collagen and neovascular membranes directly block the aqueous outflow pathway [46]. These three mechanisms are not isolated but are interrelated and may form a vicious cycle: elevated IOP exacerbates retinal ischemia, promotes the release of proangiogenic factors such as VEGF, and further worsens outflow obstruction. Therefore, intervention strategies targeting different obstructive mechanisms—goniosynechialysis for PAS, laser peripheral iridotomy for iris bombe, and combined anti‐VEGF with filtration surgery for trabecular meshwork obstruction—are critical for controlling IOP and halting disease progression.

#### 3.6.2. Neovascular Invasion of the Outflow Tract

NVG is the most aggressive form of secondary angle closure, with its pathogenesis centered on direct invasion of the angle structures by pathological neovascular membranes. The hypoxic microenvironment resulting from retinal ischemia stabilizes HIF‐1α, which in turn upregulates VEGF expression [50]. Elevated VEGF levels are strongly associated with the severity of iris and angle neovascularization [51]. These neovascular vessels originate from iris arteries, lack pericyte coverage, extend across the pupillary margin into the angle recess, and subsequently invade the trabecular meshwork, disrupting its endothelial lining and extracellular matrix structure [51].

As the disease progresses, myofibroblasts within the fibrovascular membrane contract, pulling the iris toward the cornea and forming PAS [52]. Anti‐VEGF therapy effectively induces regression of neovascularization, thereby creating safer conditions for subsequent glaucoma surgery [53]. However, although anti‐VEGF treatment inhibits VEGF‐A‐mediated endothelial proliferation, it cannot reverse established fibrotic changes; thus, the timing of therapy is critical [54]. VEGF‐A primarily promotes endothelial cell proliferation, whereas TGF‐β2 mediates myofibroblast differentiation and drives fibrosis, with each playing distinct roles at different pathological stages of NVG [55].

#### 3.6.3. Neurodegenerative Cascade

Sustained elevated IOP initiates retinal ganglion cell (RGC) degeneration through mechanisms including mitochondrial dysfunction, oxidative stress, and excitotoxicity. Elevated IOP impairs microcirculation at the optic disc, reducing oxygen delivery to RGC axons and triggering mitochondrial dysfunction. Mitochondrial dysfunction is a key driver of RGC loss in glaucoma, manifested by excessive reactive oxygen species (ROS) production, imbalanced mitochondrial dynamics (fission and fusion), and dysregulated mitophagy [56].

Oxidative stress activates proapoptotic signaling pathways such as JNK and p38 MAPK. In chronic ocular hypertension models, phosphorylation levels of the p38 MAPK pathway are elevated, and inhibition of this pathway reduces RGC apoptosis and axonal damage [57]. Glutamate excitotoxicity is another important mechanism of RGC injury. Elevated extracellular glutamate levels lead to overactivation of N‐methyl‐D‐aspartate (NMDA) receptors, causing lethal calcium influx and triggering downstream apoptotic cascades [58].

AGEs accumulate in the diabetic retina. Binding of AGEs to their receptor RAGE activates NF‐κB‐driven inflammatory pathways, which can sustain neurodegeneration even after glycemic or IOP control has been achieved. AGEs inhibit optic nerve axon elongation by inducing abnormal β‐tubulin aggregation and interfere with axonal transport and intracellular protein trafficking [59].

Notably, once initiated, neurodegeneration may progress independently of IOP levels. This phenomenon helps explain why some patients continue to experience visual field defects despite “controlled” IOP [60]. Therefore, future therapeutic strategies may need to combine IOP lowering with targeted neuroprotection, including antioxidant, mitochondrial function‐preserving, and anti‐excitotoxic interventions [60].

### 3.7. Clinical Heterogeneity and Predictive Biomarkers

#### 3.7.1. Genetic Susceptibility Loci

NVG exhibits significant interindividual variability, in which genetic susceptibility plays an important role. VEGF‐A gene polymorphisms can affect its transcriptional activity and expression levels, thereby modulating the risk of neovascularization. Studies have shown that the rs699947 (−2578C > A) polymorphism in the VEGF gene promoter is significantly associated with PDR, with A allele carriers exhibiting elevated serum VEGF levels, suggesting a potential role in PDR pathogenesis [61]. Furthermore, the VEGF gene +936C/T (rs3025039) polymorphism is associated with DR susceptibility, with meta‐analyses indicating that the T allele may increase DR risk [62]. The heterogeneity of findings across different populations suggests that genetic background may influence the strength of the association between VEGF polymorphisms and DR risk [63].

Activation of the renin‐angiotensin system (RAS) is also involved in the pathological processes of DR and NVG. The angiotensin‐converting enzyme (ACE) gene insertion/deletion (I/D) polymorphism (rs1799752) is one of the most extensively studied RAS‐related variants; however, recent meta‐analysis evidence indicates no significant association between the ACE I/D polymorphism and susceptibility to or progression of DR in patients with Type 2 diabetes mellitus [64]. Moreover, systematic reviews have also pointed out that consistent evidence for associations between RAS gene polymorphisms, including ACE I/D and AGTR1 A1166C (rs5186), and DR remains lacking [65]. These findings suggest that the role of RAS pathway gene variants in the genetic susceptibility to DR and NVG requires further validation through large‐scale, multicenter studies.

Overall, current studies on the genetic susceptibility to DR and NVG still contain many inconsistent conclusions, which may be related to differences in sample size, ethnic heterogeneity, and disease definitions. Future large‐scale genome‐wide association studies are needed to systematically identify genetic markers associated with the development and progression of NVG, thereby providing a theoretical basis for early identification of high‐risk patients and precision intervention.

#### 3.7.2. Comorbidity‐Driven Risk Stratification

Systemic comorbidities profoundly affect the course of NVG. The association between diabetic kidney disease (CKD) and retinopathy has been extensively studied. A study including 365 PDR patients showed that renal function parameters are important predictors of NVG development [66]. Patients with CKD, especially those undergoing dialysis for end‐stage renal disease, have a significantly increased risk of ocular neovascularization [67]. Uremic toxins may directly stimulate retinal endothelial proliferation by inducing endothelial cell injury and upregulating proangiogenic factors such as VEGF [68].

Hypertension, an important risk factor for DR, can promote plasma protein leakage by causing capillary mechanical stress, endothelial damage, and BRB breakdown, thereby providing a scaffold for neovascularization [69]. Studies have also found that a nondipping nocturnal blood pressure pattern is associated with severe retinal nonperfusion [31].

Vitreoretinal traction can mechanically distort retinal vessels and stimulate the production of CTGF and TGF‐β, promoting fibrovascular proliferation. Optical coherence tomography (OCT) biomarkers can effectively predict the risk of tractional retinal detachment. Studies have identified that a large area of retinal nonperfusion, extensive neovascularization, and the presence of neovascularization displaced anteriorly by vitreous traction are important predictors of tractional retinal detachment [70].

In recent years, multifactor‐integrated risk prediction models have been used for individualized management of NVG. A risk prediction model constructed using Boruta feature selection and the random forest algorithm demonstrated good predictive performance (AUC 0.87) for assessing NVG risk in PDR patients, enabling early identification of high‐risk patients and personalized intervention [66].

#### 3.7.3. Emerging Molecular Signatures

Aqueous humor analysis provides important insights into the progression from DR to NVG. The aqueous humor is a key biological sample reflecting intraocular inflammatory and proangiogenic signal gradients under retinal ischemia [71]. As the disease progresses from NPDR to PDR and NVG, stage‐specific characteristic alterations in inflammation‐ and angiogenesis‐related proteins appear in the aqueous humor, revealing the core molecular mechanisms of disease progression [72].

Multiplex immunoassay enables comprehensive assessment of the aqueous humor molecular profile and provides a basis for molecular endotyping of NVG. Proteomic studies have shown that the aqueous humor of NVG patients exhibits both activation of inflammation‐related proteins and significant upregulation of proangiogenic factors [73]. Based on aqueous humor molecular features, NVG patients can be classified into different molecular subtypes: some are dominated by inflammatory responses, showing significantly elevated inflammation‐related proteins; others are dominated by angiogenesis, characterized by enhanced expression of proangiogenic factors. Patients with different endotypes may show differential responses to therapy, suggesting that precision medicine strategies targeting the underlying biological drivers may be superior to treatments aimed solely at lowering IOP.

It should be noted that interindividual variability in the aqueous humor molecular profile is influenced by multiple factors, including the primary disease type, disease stage, and prior treatment history. With the rapid development of proteomics and bioinformatics technologies, aqueous humor molecular analysis holds promise for providing new approaches and targets for early diagnosis, risk stratification, and personalized treatment of NVG.

## 4. Treatment and Management

Based on the above mechanisms, the treatment of NVG faces unique challenges: anti‐VEGF therapy alone is insufficient to block fibrosis and inflammation. The following sections discuss the limitations of current treatment paradigms and potential combination strategies.

### 4.1. Restructuring Multimodal Treatment Strategies

#### 4.1.1. Limitations of Anti‐VEGF Monotherapy

Although anti‐VEGF monotherapy effectively promotes neovascular regression, it has significant limitations in controlling fibrosis and inflammation. Preclinical and clinical studies have shown that in the aqueous humor of PDR patients, anti‐VEGF treatment leads to a marked decrease in VEGF levels while concurrently increasing CTGF levels, resulting in an elevated CTGF/VEGF ratio [74]. The imbalance between VEGF and CTGF is considered a key molecular mechanism underlying the exacerbated fibrotic response after anti‐VEGF therapy. This phenomenon has been described clinically as “Crunch syndrome”—where, despite suppression of neovascularization, contraction of fibrovascular membranes leads to increased retinal traction and even tractional retinal detachment [75].

Moreover, anti‐VEGF monotherapy fails to effectively suppress intraocular inflammation. Studies have shown that levels of inflammatory factors such as IL‐6 and MCP‐1 in the aqueous humor of NVG patients remain persistently elevated and participate in driving the fibrotic process [76]. Therefore, anti‐VEGF monotherapy is inadequate for addressing the complex multifactorial pathogenesis of NVG. OCT observations have demonstrated that even when IOP is controlled and neovascularization has regressed, fibrovascular membranes can continue to thicken and contract.

The above evidence indicates that although anti‐VEGF therapy serves as a key initial treatment for NVG, it cannot reverse established fibrotic changes nor block inflammation‐driven pathological processes [51]. Thus, the limitations of anti‐VEGF monotherapy highlight the need for multimodal strategies that simultaneously target angiogenesis, inflammation, and fibrosis to achieve effective management of NVG [55].

#### 4.1.2. Synergistic Combination Therapy Strategies

Glucocorticoids, IL‐6 blockers, and RAS inhibitors target different nodes of the pathological network in NVG and thus hold synergistic therapeutic potential.

Glucocorticoids inhibit the NF‐κB and AP‐1 signaling pathways, downregulating proangiogenic and inflammatory factors such as VEGF, IL‐6, and TNF‐α. Simultaneously, they suppress TGF‐β1‐induced myofibroblast transformation by blocking Smad3 signaling—an antifibrotic effect not achieved by anti‐VEGF agents [77]. In clinical practice, glucocorticoids are often used as an adjunct to anti‐VEGF therapy for DR [78].

IL‐6 is a central regulator of the acute‐phase inflammatory response and a potent inducer of VEGF and TGF‐β. Elevated aqueous humor IL‐6 levels strongly correlate with the severity of NVG fibrosis. Preclinical studies have shown that the IL‐6 receptor antagonist tocilizumab reduces pathological angiogenesis and collagen deposition in ischemic retina by modulating macrophage polarization and inhibiting the STAT3/VEGF axis [79].

RAS inhibitors—specifically the angiotensin II receptor blocker losartan—suppress TGF‐β/Smad3 signaling, thereby reducing collagen synthesis and extracellular matrix production in fibroblasts, while also protecting retinal structure in diabetic models. Animal studies demonstrate that losartan treatment lowers the risk of fibrosis by downregulating TGF‐β expression and improving collagen organization [80].

The synergistic effect of these three drug classes arises from their multinode blockade of pathological pathways: glucocorticoids suppress upstream inflammatory signals, IL‐6 blockers interrupt the cytokine cascade, and RAS inhibitors antagonize TGF‐β‐driven fibrosis. Given that NVG involves multiple pathological mechanisms—angiogenesis, inflammation, and fibrosis—monotherapy with anti‐VEGF agents cannot achieve full disease control. More clinical studies are needed to validate the efficacy and safety of these combination strategies [54].

#### 4.1.3. Novel Antifibrotic Agents

Losartan, pirfenidone, and integrin antagonists provide targeted strategies to prevent the fibrotic sequelae of NVG.

Losartan inhibits angiotensin II‐mediated activation of the TGF‐β1 signaling pathway and has demonstrated antifibrotic activity in ocular fibrosis models [81]. Studies have shown that topical losartan is safe and effective in preventing and treating corneal stromal fibrosis by regulating intracellular TGF‐β signaling through inhibition of ERK phosphorylation [82].

Pirfenidone, a pleiotropic agent approved for idiopathic pulmonary fibrosis, exerts antifibrotic effects by inhibiting TGF‐β1 synthesis, blocking PDGF/FGF signaling, and scavenging ROS [83]. In experimental models of bleb fibrosis after glaucoma filtering surgery, pirfenidone together with nintedanib and rapamycin has shown promising antifibrotic activity [55].

Integrin antagonists target the mechanotransduction pathways critical for myofibroblast differentiation. RGD‐containing peptides competitively bind to integrin receptors with RGD‐containing matrix proteins, thereby blocking downstream signaling pathways [84]. Studies have confirmed that the β1‐integrin/FAK/Akt signaling axis plays an important role in fibrosis and scarring in various tissues [85]. In vitro experiments demonstrate that an RGD peptide hydrogel suppresses the expression of β1‐integrin, FAK, and Akt in Tenon’s capsule fibroblasts, offering a promising approach to prevent scarring of the glaucoma filtration channel [84].

Combining these novel antifibrotic agents with anti‐VEGF therapy could theoretically shift the treatment paradigm for NVG from reactive vascular suppression to proactive fibrosis prevention. Nevertheless, the clinical efficacy and safety of such combinations remain to be validated in large‐scale prospective clinical studies [54].

### 4.2. Unmet Research Priorities and Future Directions

Despite the advances outlined above, significant gaps remain in NVG research. The following sections discuss priority research needs.

#### 4.2.1. Limitations of Animal Models

Existing animal models cannot fully reproduce the triad of human NVG: chronic ischemia‐driven angiogenesis, dense fibrovascular membrane formation, and secondary angle closure. An ideal NVG model should simultaneously exhibit ischemic retinopathy, iris neovascularization, and angle neovascularization; however, current models still struggle to recapitulate this entire process [86].

The rodent oxygen‐induced retinopathy (OIR) model is one of the most commonly used tools for studying retinal neovascularization and can simulate certain features of retinopathy of prematurity and proliferative DR [87]. Nevertheless, the OIR model mainly produces superficial retinal neovascularization that typically regresses spontaneously within a specific postnatal time window. Due to significant anatomical differences in the anterior segment between rodents and primates, this model rarely progresses to anterior segment neovascularization [88]. The laser‐induced retinal vein occlusion (RVO) model has been successfully established in various animal species and is used to investigate the pathological mechanisms of ischemic retinopathy [89]. Although this model leads to retinal nonperfusion and neovascularization, it also fails to generate iris neovascularization or angle fibrovascular membranes in rodents; moreover, the extent and duration of fibrosis differ considerably from human NVG.

Intravitreal injection of VEGF in nonhuman primates can induce iris neovascularization and even NVG [90]. This model demonstrates the sufficiency of VEGF in driving iris neovascularization; however, the resulting neovascular membranes are mostly inflammatory and exudative, lacking the dense, myofibroblast‐rich, contractile fibrovascular membranes characteristic of human NVG, and they rarely leave permanent angle adhesions after vascular regression.

A more ideal model would need to integrate sustained ischemia, chronic inflammation, and a profibrotic microenvironment. The TGF‐β2 overexpression model can be used to study the interaction between fibrosis and elevated IOP, but it mainly reflects trabecular meshwork fibrosis as seen in primary open‐angle glaucoma and cannot fully reproduce the multicellular complexity of the fibrovascular tissue originating from the ischemic retina in human NVG [91]. Combining panretinal photocoagulation with anti‐VEGF therapy in diabetic rodents can, to some extent, mimic the TGF‐β‐driven fibrosis that occurs after clinical VEGF blockade; however, such interventions often cause extensive retinal damage, making it difficult to precisely control the site and degree of fibrosis.

Future development of NVG animal models should prioritize endpoints beyond the area of neovascularization, including dynamic monitoring of the aqueous humor cytokine profile, quantification of PAS by anterior‐segment OCT, and biomechanical characterization of fibrovascular membranes, in order to better simulate the therapeutic challenges of human NVG.

#### 4.2.2. Advanced Imaging Integration

OCT angiography (OCTA) and high‐resolution anterior segment OCT (AS‐OCT) offer noninvasive tools for early detection of NVG [92]. AS‐OCT‐based models can identify the presence and extent of PAS and detect early angle closure before IOP rises [93]. When OCTA shows neovascularization without PAS on AS‐OCT, a therapeutic window for medical treatment may exist; when AS‐OCT reveals dense fibrovascular membranes with no flow signal on OCTA, anti‐VEGF is no longer effective and surgical planning is indicated. Key barriers include the lack of normative databases, susceptibility to motion artifacts, and the absence of validated grading systems for NVG‐specific findings [94]. Future directions may focus on AI‐based automated analysis of OCTA/AS‐OCT data to generate risk scores for angle closure or fibrosis progression and integration with aqueous humor molecular profiling as composite endpoints in clinical trials [95].

### 4.3. Toward an Integrated Systemic Approach to NVG Management

NVG management should not be confined to ocular treatment alone but must also incorporate systemic metabolic status and multidisciplinary collaboration.

#### 4.3.1. Multidisciplinary Collaboration

NVG management requires a shift from isolated subspecialty care to a multidisciplinary approach integrating endocrinology, retina, and glaucoma specialties. Existing studies support the establishment of unified protocols and automated referral mechanisms: when a retinal specialist identifies high‐risk PDR patients with extensive retinal nonperfusion, endocrinology consultation for glycemic control optimization and glaucoma evaluation (AS‐OCT/gonioscopy) should be triggered simultaneously [96]. Standardized electronic health record dashboards displaying HbA1c trends, nonperfusion areas, and PAS extent in real time enable proactive intervention. The multidisciplinary model is expected to shorten intervention times and improve visual outcomes [96].

#### 4.3.2. NVG as a Manifestation of Systemic Metabolic Dysregulation

NVG represents not only an ocular emergency but also a local manifestation of systemic endothelial dysfunction, chronic inflammation, and metabolic stress. Serum biomarker studies have shown elevated LRG1 and FGF‐21 levels in NVG patients, which positively correlate with intraocular VEGF and TNF‐α levels. Systemic inflammatory markers including neutrophil‐to‐lymphocyte ratio and systemic immune‐inflammation index are significantly elevated in NVG patients, suggesting the involvement of systemic inflammation in its pathogenesis [97]. Proteomic studies have further confirmed that the aqueous humor protein profile exhibits characteristic changes during DR progression, including activation of inflammatory pathways and ECM remodeling, reinforcing the intrinsic link between ocular pathology and systemic status [71].

NVG frequently coexists with systemic vascular conditions such as diabetic kidney disease, peripheral artery disease, and heart failure. DR and cardiovascular disease are interconnected through shared mechanisms, including oxidative stress, inflammation, endothelial dysfunction, and the AGEs‐RAGE axis, positioning DR as a biomarker of systemic vascular risk [98]. Interventions targeting systemic redox balance, such as omega‐3 fatty acids, have demonstrated potential in reducing the risk of diabetic microvascular complications [99]. Optimizing nocturnal blood pressure to maintain ocular perfusion pressure and prevent watershed infarction also carries significant clinical implications [100].

#### 4.3.3. Vision for “Pre‐NVG” Screening

The future of NVG management lies in proactive identification of “pre‐NVG” individuals—those at risk before clinical neovascularization become apparent—through multiomics signatures and subtle imaging changes. Aqueous humor proteomics has revealed characteristic molecular alterations during DR progression to NVG, offering potential targets for early screening [71]. Aberrant kynurenine pathway activity reflects mitochondrial dysfunction, and its metabolite alterations are associated with diabetes and glaucoma progression [101]. AI‐enhanced OCT‐A can detect microvascular flow voids, while AS‐OCT quantifies subtle angle narrowing, enabling structural warning [102]. An integrated risk score combining multiomics data with quantitative imaging metrics holds promise for transforming NVG from a devastating complication into a preventable disease.

## 5. Conclusion

NVG in DR represents the end‐stage outcome of systemic metabolic failure, chronic inflammation, and maladaptive tissue remodeling. The core cascade involves microvascular occlusion induced by hyperglycemia, stabilization of HIF‐1*α* due to retinal hypoxia, and VEGF/PlGF‐driven angiogenesis. In the setting of deficient endogenous antiangiogenic factors such as PEDF and uncontrolled innate immune activation, neovascular complexes invade the iris and angle. Dysregulated TGF‐β‐mediated myofibroblast differentiation and extracellular matrix deposition render anti‐VEGF monotherapy ineffective, leading to irreversible PAS, trabecular meshwork obstruction, and severe ocular hypertension. This in turn triggers secondary optic neuropathy through mechanical compression and mechanisms including mitochondrial dysfunction, oxidative stress, and excitotoxicity.

Clinical heterogeneity arises from genetic susceptibility, comorbidities, and molecular signatures defining distinct endotypes. Current management requires multimodal strategies targeting angiogenesis, inflammation, and fibrosis—such as corticosteroids, IL‐6 blockers, RAS inhibitors, and novel antifibrotic agents—while addressing research gaps including longitudinal aqueous humor profiling, representative animal models, and advanced imaging applications.

Effective prevention of NVG calls for an integrated systemic approach that redefines NVG as a manifestation of systemic metabolic dysregulation, promotes multidisciplinary collaboration, and implements “pre‐NVG” screening through multiomics risk stratification, thereby enabling proactive, precision‐guided intervention before irreversible blindness occurs.

## Author Contributions

Xuan Wang and Siyan Liu conceived the review, conducted the primary literature search, performed data curation and evidence synthesis, and drafted the manuscript. Qi Wu, Yuxin Lv, Shuangshuang Yi, and Bowen Shi contributed to targeted literature retrieval, reference verification, figure preparation, and manuscript revision. Zhengzheng Wu and Jing Yan secured funding, supervised the project, and critically revised the manuscript for important intellectual content.

## Funding

This study was supported by the High‐Level Program of China Academy of Traditional Chinese Medicine Eye Hospital (Grant GSP2‐17, GSP3‐13).

## Disclosure

The funder was not involved in the study design, data collection, analysis, interpretation, or manuscript writing. The literature search, manuscript writing, and all intellectual content were produced by the authors, who reviewed and verified all machine‐generated suggestions and take full responsibility for the final text.

## Ethics Statement

The review of this article did not involve any clinical data, human participants, or experimental animals. All data were extracted from publicly available published literature, and no personal privacy issues were involved. As this study did not involve any direct interaction with human subjects or the use of identifiable data, ethical approval was not required.

## Conflicts of Interest

The authors declare no conflicts of interest.

## Data Availability

This is a review article and no original data were generated.
